# Health-related quality of life in patients with autoimmune hepatitis

**DOI:** 10.1007/s11136-021-02850-0

**Published:** 2021-05-12

**Authors:** Maurice Michel, Francesca Spinelli, Annette Grambihler, Christian Labenz, Michael Nagel, Leonard Kaps, Yvonne Huber, Peter R. Galle, Marcus-Alexander Wörns, Jörn M. Schattenberg

**Affiliations:** 1 Metabolic Liver Research Program, I. Department of Medicine, University Medical Centre Mainz, Mainz, Germany; 2 I. Department of Medicine, University Medical Centre Mainz, Mainz, Germany

**Keywords:** AIH, Biochemical remission, HRQL, CLDQ, EQ-5D-5L

## Abstract

**Background:**

Autoimmune hepatitis (AIH) is a rare chronic liver disease. Impaired health-related quality of life (HRQL) contributes to the overall disease burden. At current, only limited data related to the impact of treatment response on HRQL are available.

**Objective:**

The aim of the study was to determine the impact of biochemical remission on HRQL.

**Methods:**

Patients with AIH were prospectively enrolled between July 2018 and June 2019. A liver disease-specific tool, the chronic liver disease questionnaire (CLDQ) and the generic EQ-5D-5L were used to quantify HRQL. Treatment response was assessed biochemically by measurement of immunoglobulin G, ALT and AST. The cohort was divided into two groups according to their biochemical remission status in either complete vs. incomplete remission. Clinical as well as laboratory parameters and comorbidities were analysed using univariable and multivariable analysis to identify predictors of poor HRQL.

**Results:**

A total of 116 AIH patients were included (median age: 55; 77.6% female), of which 9.5% had liver cirrhosis. In this cohort, 38 (38.4%) showed a complete and 61 (61.6%) an incomplete biochemical remission at study entry. The HRQL was significantly higher in patients with a complete as compared to an incomplete biochemical remission (CLDQ overall score: 5.66 ± 1.15 vs. 5.10 ± 1.35; *p* = 0.03). In contrast, the generic EQ-5D-5L UI-value was not different between the groups. Multivariable analysis identified AST (*p* = 0.02) and an incomplete biochemical remission (*p* = 0.04) as independent predictors of reduced HRQL (CLDQ total value).

**Conclusion:**

Patients with a complete biochemical remission had a significantly higher HRQL. Liver-related quality of life in patients living with AIH is dependent on the response to immunosuppressive treatment.

**Supplementary Information:**

The online version contains supplementary material available at 10.1007/s11136-021-02850-0.

## Introduction

Autoimmune hepatitis (AIH) is a rare, chronic liver disease characterized by chronic inflammation targeting hepatocytes with a prevalence ranging from 16 to 18 cases per 100,000 inhabitants in Europe [1]. If left untreated, AIH can progress to end stage liver disease or acute liver failure with liver transplantation being required [2, 3]. Immunosuppressive therapy has significantly improved the prognosis of patients with AIH [4]. The combination of corticosteroids with azathioprine is recommended for first line treatment. Frequently, tapering of immunosuppressive therapy over time can be successfully performed without losing remission [5, 6]. By preventing recurrent flares, disease progression can be halted in most patients, although long-term mortality still remains higher compared to the general population [7]. In the management of AIH, biochemical remission defined by normalization of ALT, AST and immunoglobulin G (IgG) is considered a complete response to treatment [8, 9].

Beyond biochemical remission, reported or perceived health is critical for the management of patients with AIH, and health-related quality of life (HRQL) is an integral part of the general concept of being healthful. HRQL—the determination of how a patient feels and functions—can be measured by different instruments in defined domains that include general health or disease-specific health aspects. HRQL is potentially influenced by disease activity, but also adverse effects from treatment or comorbidities. In patients with AIH, an increased prevalence of anxiety and major depressive symptoms have been observed [10]. While an effect of these comorbidities on HRQL appears obvious, depression and anxiety are also associated with a greater degree of non-adherence to immunosuppressive therapy [11] and thus can accelerate disease progression and the burden of disease. Adverse effects from the use of corticosteroids can also potentially impair HRQL in AIH [12]. In a Japanese study exploring 265 patients with AIH, a negative impact of corticosteroid use on the subdomain ‘worry’—but not overall HRQL scores—was detected [13]. Studies that explored budesonide did not find a difference for the use of budesonide over corticosteroids with regards to HRQL [14].

Currently, there is only limited data available related to the impact of biochemical remission on HRQL in AIH. Yet, no study has focussed on correlating clinical and biochemical parameters with HRQL. Hence, the aim of this study was to analyse HRQL using a liver disease-specific tool, compare these to generic measures of quality of life and determine the effects of biochemical remission and identify surrogate markers of HRQL.

## Materials and methods

### Study population and population subtypes

A total of 116 patients with AIH were enrolled between July 2018 and July 2019 in this non-interventional and cross-sectional cohort study after informed consent was obtained at the outpatient clinic of the Metabolic Liver Research Program of the University Medical Centre Mainz in Germany. Inclusion criteria were AIH and AIH-overlap according to the EASL clinical practice guidelines [8]. AIH-overlap included patients with primary biliary cholangitis (PBC) or primary sclerosing cholangitis (PSC) in addition to the diagnosis of AIH. For inclusion, patients had to be at least 18 years of age. Patients with an expected life expectancy of < 6 months were not included, in particular patients with a history of hepatocellular carcinoma (HCC) or malignancies were not included. Coexisting liver disease other than AIH or AIH-overlap (i.e. viral hepatitis) were excluded. Laboratory values were obtained on the day of inclusion. Medical and treatment history were recorded and available from the electronic health care records. Immunosuppressive therapy was recorded at the time of the HRQL assessment by the treating hepatologists. To investigate the impact of actual treatment and response on HRQL, only current treatments were considered.

### Definition of biochemical remission

Biochemical remission at study entry was defined as either complete (normalization of ALT, AST and IgG) or incomplete (elevation of one or two, or persistently elevated levels of the surrogates: ALT, AST and IgG) according to published criteria [15]. Complete normalization had to be maintained for at least 6 months before considering complete remission. Due to missing blood values, 17 patients were excluded for the final comparison between complete vs. incomplete biochemical remission (*n* = 99). The upper limit of normal (ULN) was defined as follows according to the reference ranges of the Institute of Clinical Chemistry and Laboratory Medicine at University Medical Centre Mainz: ALT in males ≤ 50 U/l, ALT in females ≤ 35 U/l, AST in males ≤ 35 U/l, AST in females ≤ 31 U/l, IgG ≤ 16.0 g/L irrespective of sex. Furthermore, the ULN of the remaining laboratory values was as follows: ALP in males ≤ 138, ALP in females ≤ 111 U/l, gGT in males ≤ 64 U/l, gGT in females ≤ 36 U/l, bilirubin ≤ 1.2 mg/dl irrespective of sex.

### Health-related quality of life (HRQL) assessment

HRQL was assessed by the validated German version of the Chronic Liver Disease Questionnaire (CLDQ) and the European Quality-of-Life 5-Dimension 5-Level (EQ-5D-5L) questionnaire under standardized conditions. The CLDQ compromises a total of 29 items and it is divided into 6 subscale scores (abdominal symptoms, fatigue, systemic symptoms, activity, emotional functioning, worry) and an overall score. The results of the scores are presented on a scale from 1 to 7, with 1 indicating worst HRQL (bad) and 7 indicating best HRQL (good). The CLDQ is more specific regarding liver related health issues [16]. The EQ-5D-5L represents a general assessment of the quality of life [17], which has also been used in AIH previously and other liver related pathologies [12]. It consists of five dimensions, including mobility, self-care, usual activities, pain/discomfort and anxiety/depression with each dimension having five response levels: no problems, slight problems, moderate problems, severe problems and unable to/extreme problems. The response levels are represented by numbers from 1 to 5, with 1 indicating “no problems” and 5 indicating “extreme problems”. The EQ visual analogue scale (VAS) is part of the EQ-5D-5L score and records the respondent’s overall current health, ranging from 0 (the worst health you can imagine) to 100 (the best health you can imagine). From these five subdimensions, the utility index (UI) was calculated. In order to calculate the UI, the EQ-5D-5L value set for Germany was used [18]. In this study, the calculated UI ranges from − 0.21 (worst health) to 1 (best health). Higher values on both VAS and UI indicate a higher HRQL. In contrast, a higher score on the subdomains of EQ-5D-5L is associated with a lower HRQL. Both questionnaires were completed by all enrolled patients (*n* = 116) during the visit at the outpatient study centre.

### Ethics

All patients provided written informed consent. The study was conducted according to the ethical guidelines of the 1975 Declaration of Helsinki (6th revision, 2008). The study protocol was approved by the ethics committee of the Landesärztekammer Rhineland-Palatine on 25th May 2010 with amendment on 07th May 2013 (Nr. 873.199.10 7208).

### Statistical analysis

Descriptive analysis of data is expressed as either mean or median with standard deviations or interquartile ranges (IQR). To compare groups and to calculate differences between two groups with quantitative values, Mann–Whitney *U* rank test was used. For the comparison of two or more patient groups a Chi-square test was applied. All tests were two-tailed, statistically significant values were defined as *p* < 0.05. Univariable regression analysis was used to examine associations between two variables. All variables with *p* < 0.1 and based on clinical importance were then included into a multivariable linear regression model. To avoid multicollinearity, the variables AST and incomplete remission were independently analysed in two multivariable models, respectively. Because the data analysis was exploratory, no adjustment for multiple testing was performed. Due to the large number of tests, *p*-values should be interpreted with caution and in connection with effect estimates. For all data analysis and statistical tests, IBM SPSS Statistic Version 23.0 (Armonk, NY: IBM Corp.) was used.

## Results

### Study population

A total of 116 patients with AIH were enrolled prospectively. The majority of patients were female (*n* = 90, 77.6%) with a median age of 55 years (IQR 41; 64) at inclusion. Baseline characteristics are summarized in Table 1. In the entire cohort, median ALT (30.5 U/I (IQR 21; 44.25)) was below ULN, while median AST (33 U/I (IQR 26; 42)) and gGT (38 U/I (IQR 22.5; 84.5) were slightly above the ULN. Likewise, median bilirubin (total bilirubin: 0.72 mg/dl; IQR 0.50, 1.01) and alkaline phosphatase (ALP; 87 U/I; IQR 64; 117) were within the ULN. A total of 19 (16.4%) patients were diagnosed with AIH-overlap syndrome with primary biliary cholangitis (PBC) in 11 (9.5%) and primary sclerosing cholangitis (PSC) in 8 (6.9%) patients. Compensated liver cirrhosis was present in 11 patients (9.5%). Most patients were treated with azathioprine (87%), while 58.8% received corticosteroids. A complete biochemical remission was seen in 38.4% (*n* = 38) of patients. In turn, 61.6% (*n* = 61) showed an incomplete biochemical remission, as indicated by only partial normalization (either one or two of ALT/AST and IgG) or persistently elevated blood levels of ALT, AST and IgG.

**Table 1:** Clinical characteristics, demographic data and differences between complete and incomplete biochemical remission

Variables	Total cohort (n = 116)	Complete remission (*n* = 38)	Incomplete remission (*n* = 61)	*p*-value
	*n* (%) or median (25th; 75th)	*n* (%) or median (25th; 75th)	*n* (%) or median (25th; 75th)	
Age at inclusion	55 (41; 64)	54 (40.5; 73)	52 (41; 64)	0.18
Sex, female	90 (77.6)	31 (81.6)	46 (75.4)	0.32
Time since diagnosis (> 10 years)	48 (41.4)	17 (44.7)	27 (44.3)	0.56
Disease duration (years)	6 (2.5; 13)	8 (3.2;13.7)	6 (2; 14)	0.64
Type 2 diabetes	15 (12.9)	4 (10.5)	10 (16.4)	0.42
Arterial hypertension	28 (24.1)	10 (26.3)	14 (22.9)	0.70
Laboratory				
ALT (U/l) *n* = 106	30.5 (21; 44.25)	22.5 (14.5; 29.5)	38 (31; 74)	<** 0**.**001**
AST (U/I) *n* = 107	33 (26; 42)	26 (22; 28.7)	39 (34; 58)	<** 0**.**001**
ALP (U/l) *n* = 93	87 (64; 117)	69.5 (57.7; 68.2)	101 (73; 132)^a^	**0**.**004**
gGT (U/l) *n* = 105	38 (22.5; 84.5)	24.5 (20.7; 29.7)	66 (29; 87)	<** 0**.**001**
Total bilirubin (g/dl) *n* = 101	0.72 (0.50; 1.01)	0.74 (0.6; 0.86)	0.63 (0.5; 0.9)	0.82
IgG (g/dl) *n* = 99	12 (10.4; 15.7)	10.2 (8.9; 12.3)	13.2 (11.5; 17.7)	<** 0**.**001**
Thrombocytes (1000/µ) *n* = 99	237 (187; 289)	238 (194; 302)	240 (187; 284)^b^	0.71
Medication				
Steroids	67 (58.8)	15 (39.5)	42 (68.9)	**0**.**004**
Prednisolone	42 (37.2)	9 (23.7)	26 (42.6)	0.11
Budesonide	25 (21.6)	6 (15.8)	16 (26.3)	0.17
Azathioprine	101 (87)	34 (89.5)	53 (86.9)	0.76
Azathioprine monotherapy	42 (36.2)	23 (60.6)	16 (26.2)	0.08
Azathioprine and steroid therapy	59 (50.8)	11 (28.9)	37 (60.7)	**0**.**001**
AIH-related comorbidities				
AIH-overlap syndrome	19 (16.4)	4 (10.6)	11 (18)	0.24
PBC	11 (9.5)	2 (5.3)	6 (9.8)	0.33
PSC	8 (6.9)	2 (5.3)	5 (8.2)	0.43
Liver cirrhosis	11 (9.5)	4 (10.5)	5 (8.2)	0.48

Data are expressed as numbers, median, percentage (%) or interquartile ranges (IQR)

*ALT* alanine-aminotransaminase, *AST* aspartat-aminotransaminase, *ALP* alkaline phosphatase, *gGT* gamma-GT, *IgG* immunoglobulin G

*p*-values refer to the comparison between complete vs. incomplete remission. Boldface indicates statistical significance. A *p*-value < 0.05 was considered statistically significant

^a^Measured in 56 patients

^b^Measured in 57 patients

### Comparison of patients with complete and incomplete biochemical remission

The median age between patients with complete and incomplete remission was comparable between both groups. Besides the surrogate markers of biochemical remission, gGT and ALP blood levels were significantly higher in the incomplete remission group. More patients with incomplete remission were on a dual therapy with azathioprine and steroids and showed a higher use of steroids in comparison to patients with a complete remission (Table 1).

### Health-related quality of life in AIH

The mean CLDQ total value in the study cohort was 5.31 ± 1.25. The subdomain fatigue of the CLDQ showed the lowest score (4.34 ± 1.66) with no significant difference between male or female. In contrast, the subdomain activity of the CLDQ showed the highest mean score with 5.84 ± 1.31 (Table 2), and it significantly differed between male and female (m: 6.37 ± 0.89 vs. f: 5.69 ± 1.37; *p* = 0.01) (Supplementary Table 1). In the EQ-5D-5L, the subdomain pain/discomfort showed the highest mean score (1.97 ± 0.98) while the domain self-care had the lowest mean score (1.23 ± 0.66). The mean VAS score and UI-value were 71.2 ± 20.5 and 0.86 ± 0.18, respectively. In women, the EQ-5D-5L UI-value and the subdomain pain/discomfort were lower compared to men. Overall, no difference in HRQL between patients with AIH and AIH-overlap syndrome were detectable (Supplementary Table 2).

**Table 2:** Mean scores of each questionnaire

Variable	Total cohort (*n* = 116)
CLDQ	
CLDQ abdominal symptoms	5.57 ± 1.50
CLDQ fatigue	4.34 ± 1.66
CLDQ systemic symptoms	5.45 ± 1.31
CLDQ activity	5.84 ± 1.31
CLDQ emotional function	5.10 ± 1.38
CLDQ worry	5.55 ± 1.39
CLDQ total value	5.31 ± 1.25
EQ-5D-5L	
EQ-5D-5L mobility	1.49 ± 0.88
EQ-5D-5L self-care	1.23 ± 0.66
EQ-5D-5L usual activities	1.60 ± 0.95
EQ-5D-5L pain/discomfort	1.97 ± 0.98
EQ-5D-5L anxiety/depression	1.63 ± 0.94
EQ-5D-5L VAS	71.2 ± 20.5
EQ-5D-5L UI-value	0.86 ± 0.18

Data are expressed as means with standard deviation

### Health-related quality of life in complete vs. incomplete biochemical remission

**Fig. 1:**
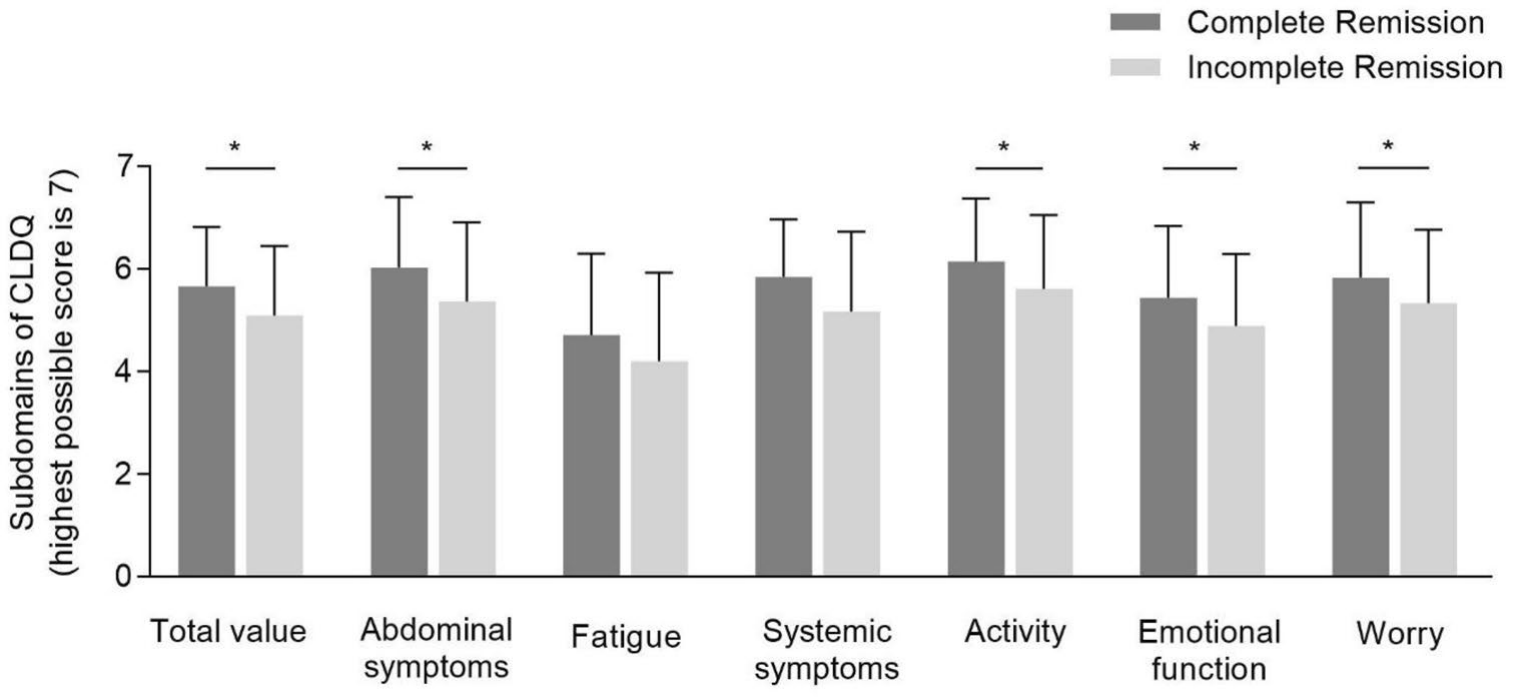
Scores of CLDQ total value and its respective subdomains in complete and incomplete biochemical remission. Scores are represented as mean values ± standard deviation. A *p*-value < 0.05 is considered statistically significant (*p < 0.05). Please note: A higher value on CLDQ indicates a better HRQL with 7 being the highest possible score to obtain

Next, the cohort was divided into two groups—complete vs. incomplete biochemical remission—according to the blood levels of ALT, AST and IgG at the day of presentation. The total CLDQ score was significantly higher in complete remission (CR) as compared to incomplete remission (IR) (CR: 5.66 ± 1.15 vs. IR: 5.10 ± 1.35, *p* = 0.03). The CLDQ-subdomains abdominal symptoms (CR: 6.03 ± 1.38 vs. IR: 5.36 ± 1.53, *p* = 0.01), activity (CR: 6.15 ± 1.23 vs. IR: 5.61 ± 1.44, *p* = 0.03), emotional function (CR: 5.44 ± 1.40 vs. IR: 4.88 ± 1.41, *p* = 0.04) and worry (CR: 5.83 ± 1.47 vs. IR: 5.34 ± 1.43, *p* = 0.03) were significantly higher in the group with complete biochemical remission (Fig. 1). Although the subdomain fatigue had the lowest score overall, no significant difference was seen between patients with complete or incomplete biochemical remission.

**Fig. 2:**
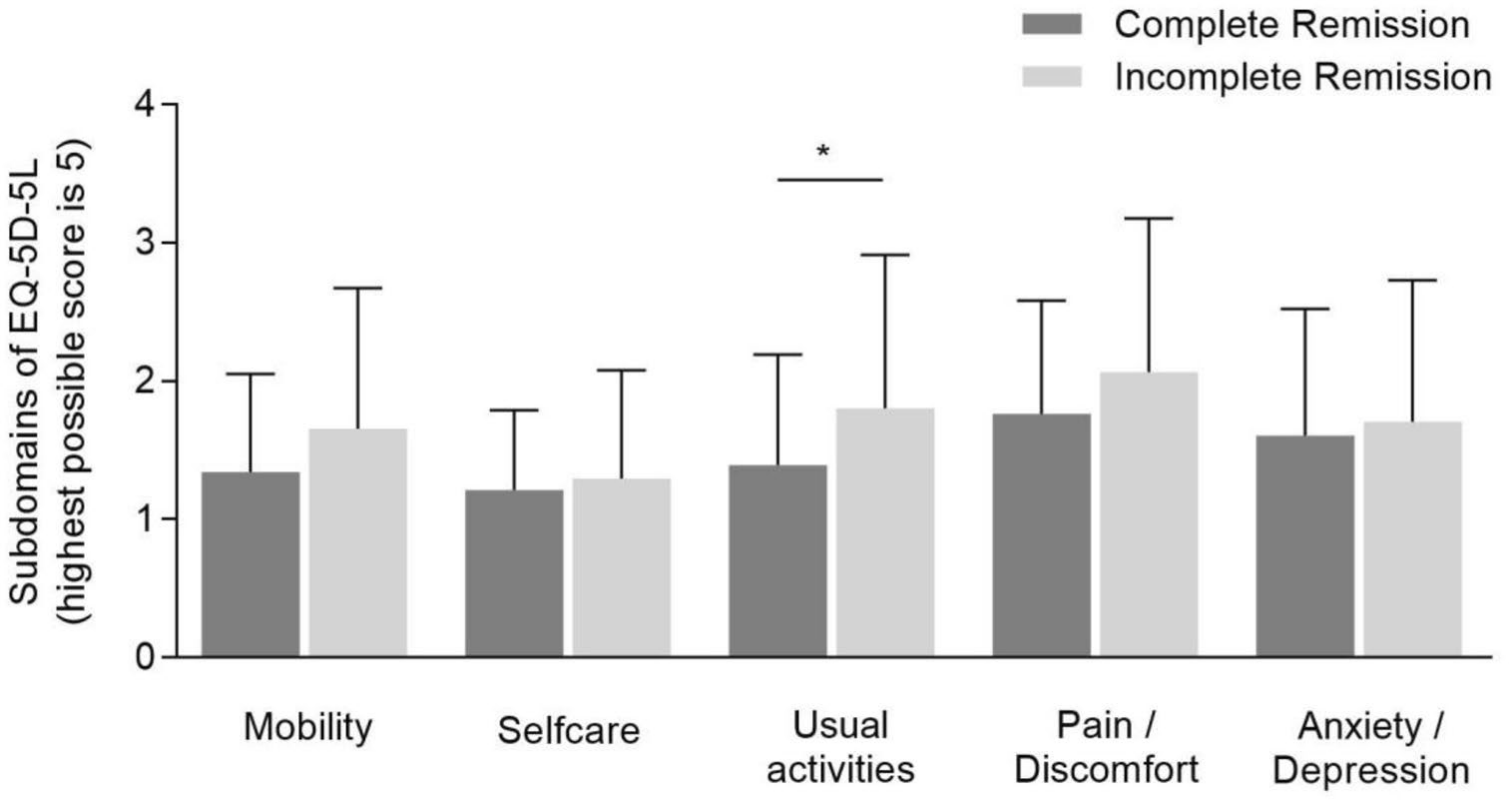
Scores of EQ-5D-5L and its respective subdomains in complete and incomplete biochemical remission. Scores are represented as mean values ± standard deviation. A *p*-value < 0.05 is considered statistically significant (*p < 0.05). Please note: A higher value on EQ-5D-5L subdomains indicates a worse HRQL with 5 being the highest possible score to obtain

Using the generic HRQL EQ-5D-5L questionnaire, only the subdomain ‘usual activities’ showed a significant difference between the two groups (CR: 1.34 ± 0.71 vs. IR: 1.80 ± 1.2, *p* = 0.03) (Fig. 2). Patients with an incomplete remission exhibited a numerically lower VAS of 70 ± 21.3 and lower UI-value of 0.83 ± 0.22, with, however, no statistically significant difference. The complete statistical analysis of both questionnaires is provided in Supplementary Table 3.

### Predictors of poor health-related quality of life in AIH

In a univariable analysis, an incomplete remission (*β*: − 0.21; 95% CI − 0.43, − 0.02), blood levels of AST (*β*: − 0.21; 95% CI − 0.41, − 0.02) and type 2 diabetes (*β*: − 0.21; 95% CI − 0.39, − 0.03) were associated with an impaired HRQL. In a multivariable linear regression analysis, AST (*β*: − 0.22; 95% CI − 0.42, − 0.03) and incomplete remission (*β*: − 0.21; 95% CI − 0.38, − 0.02) were the two factors that remained independently associated with a worse HRQL (Table 3). In turn, type 2 diabetes (*β*: − 0.37; 95% CI − 0.54, − 0.20) was the only factor to be associated with an impaired HRQL as captured by EQ-5D-5L UI-value in an univariable analysis. In a multivariable linear regression analysis, type 2 diabetes (*β*: − 0.35; 95% CI − 0.56, − 0.15) remained independently associated with a worse HRQL (Table 4).

**Table 3:** Uni- and multivariable analyses of clinical and laboratory parameters with CLDQ total value

Variable	CLDQ total value								
	Univariable analysis			Multivariable analysis^a^			Multivariable analysis^b^		
	*β*	95% CI	*p*	*β*	95% CI	*p*	*β*	95% CI	*p*
Sex	− 0.11	− 0.31, 0.06	0.19	− 0.13	− 0.32, 0.07	0.19	− 0.08	− 0.34, 0.06	0.16
Age at study entry	− 0.07	− 0.27, 0.10	0.37						
Disease duration	− 0.07	− 0.28, 0.11	0.37						
Time since diagnosis (> 10 years)	− 0.16	− 0.34, 0.02	0.09	− 0.14	− 0.34, 0.04	0.13	− 0.14	− 0.34, 0.06	0.16
AST	− **0**.**21**	− **0**.**41**, − **0**.**02**	**0**.**03**	− **0**.**22**	− **0**.**42**, − **0**.**03**	**0**.**02**			
ALT	− 0.05	− 0.25, 0.15	0.64						
gGT	− 0.09	− 0.29, 0.11	0.38						
IgG	0.01	− 0.21, 0.22	0.95						
Steroids	− 0.09	− 0.28, 0.10	0.33						
Azathioprine monotherapy	0.03	− 0.16, 0.22	0.75						
Azathioprine and steroid therapy	− 0.10	− 0.30, 0.10	0.32						
Incomplete remission	− **0**.**21**	− **0**.**43**, − **0**.**02**	**0**.**03**				− **0**.**21**	− **0**.**38**, − **0**.**02**	**0**.**04**
Liver cirrhosis	− 0.11	− 0.29, 0.08	0.25						
AIH-overlap	− 0.02	− 0.20, 0.17	0.89						
Type 2 diabetes	− **0**.**21**	− **0**.**39**, − **0**.**03**	**0**.**02**	− 0.17	− 0.36, 0.02	0.08	− 0.18	− 0.38, 0.02	0.08
Arterial hypertension	− 0.15	− 0.33, 0.03	0.11						

Sex: 1 for male, 2 for female; Time since diagnosis (> 10 years): 1 for No, 2 for Yes; Steroids 1 for No, 2 for Yes; Azathioprine monotherapy: 1 for No, 2 for Yes; Azathioprine and steroid therapy: 1 for No, 2 for Yes; Incomplete remission: 1 for No, 2 for Yes; Liver Cirrhosis: 1 for No, 2 for Yes; AIH-Overlap: 1 for No, 2 for Yes; Type 2 Diabetes: 1 for No, 2 for Yes; Arterial Hypertension: 1 for No, 2 for Yes; ALT, Alanine-Aminotransaminase; AST, Aspartat-Aminotransaminase; gGT, Gamma-GT; IgG, Immunoglobulin G

At first, univariable analysis of data was done. With all factors showing a *p*-value < 0.1 and the clinical parameter ‘sex’, a multivariable linear regression model was built. Confidence Interval (CI) and Beta (*β*) show standardized values, respectively. Boldface indicates statistical significance. A *p*-value < 0.05 was considered statistically significant

^a^Multivariable linear regression model including the variables: sex, time since diagnosis, AST, type 2 diabetes

^b^Multivariable linear regression model including the variables: sex, time since diagnosis, incomplete remission, type 2 diabetes

**Table 4:** Uni- and multivariable analysis of clinical and laboratory parameters with EQ-5D-5L UI-value

Variable	UI-value					
	Univariable analysis			Multivariable analysis^a^		
	*β*	95% CI	*p*	*β*	95% CI	*p*
Sex	− 0.12	− 0.31, 0.06	0.19	− 0.03	− 0.24, 0.17	0.74
Age at study entry	− 0.10	− 0.29, 0.08	0.27			
Disease duration	− 0.01	− 0.20, 0.18	0.92			
Time since diagnosis (> 10 years)	− 0.06	− 0.25, 0.13	0.52			
AST	− 0.11	− 0.31, 0.09	0.27			
ALT	− 0.01	− 0.20, 0.20	0.99			
gGT	-0.03	− 0.23, 0.19	0.79			
IgG	0.08	− 0.13, 0.30	0.43			
Steroids	− 0.14	− 0.33, 0.05	0.14			
Azathioprine monotherapy	0.10	− 0.08, 0.29	0.28			
Azathioprine and steroid therapy	− 0.14	− 0.35, 0.06	0.16			
Incomplete remission	− 0.17	− 0.40, − 0.03	0.09	− 0.15	− 0.36, 0.04	0.12
Liver cirrhosis	− 0.03	− 0.21, 0.16	0.79			
AIH-overlap	0.09	− 0.09, 0.28	0.33			
Type 2 diabetes	− **0**.**37**	− **0**.**54**, − **0**.**20**	<** 0**.**001**	− **0**.**35**	− **0**.**56**, − **0**.**15**	**0**.**001**
Arterial hypertension	− 0.15	− 0.38, 0.03	0.10	− 0.08	− 0.22, 0.17	0.42

Sex: 1 for male, 2 for female; Time since diagnosis (> 10 years): 1 for No, 2 for Yes; Steroids 1 for No, 2 for Yes; Azathioprine monotherapy: 1 for No, 2 for Yes; Azathioprine and steroid therapy: 1 for No, 2 for Yes; Incomplete remission: 1 for No, 2 for Yes; Liver Cirrhosis: 1 for No, 2 for Yes; AIH-Overlap: 1 No, 2 for Yes; Type 2 Diabetes: 1 for No, 2 for Yes; Arterial Hypertension: 1 for No, 2 for Yes; ALT, Alanine-Aminotransaminase; AST, Aspartat-Aminotransaminase; gGT, Gamma-GT; IgG, Immunoglobulin G

At first, univariable analysis of data was done. With all factors showing a *p*-value < 0.1 and the clinical parameter ‘sex’, a multivariable linear regression model was built. Confidence Interval (CI) and Beta (*β*) show standardized values, respectively. Boldface indicates statistical significance. A *p*-value < 0.05 was considered statistically significant

^a^Multivariable linear regression model including the variables: sex, incomplete remission, type 2 diabetes, arterial hypertension

## Discussion

In this study, we assessed HRQL using two validated questionnaires—the liver-specific CLDQ and the generic EQ-5D-5L—in patients with AIH. Each questionnaire is validated in German and carries several subdomains to address different aspects of a patients’ well-being. The central finding of the current analysis is that patients with a complete biochemical remission have a higher HRQL in comparison to an incomplete remission in this cohort, when using a liver disease-specific tool. While the CLDQ overall score as well as several subdomains exhibited a positive correlation with biochemical remission, no correlation with the general HRQL tool—EQ-5D-5L—was detectable. One important aspect is that the CLDQ was designed to capture liver-specific aspects of the quality of life, which are not assessed by the generic/general questionnaires [16]. Additionally, we observed a significant correlation of several liver related blood and clinical parameters with the total value of CLDQ, but not the UI-value of EQ-5D-5L.

The CLDQ overall score was 5.31 among all AIH patients. Interestingly, this overall score was comparable to patients with compensated cirrhosis and covert hepatic encephalopathy that were assessed in the same outpatient setting [19] and also comparable to a Japanese study that compared AIH with healthy controls [13]. Furthermore, fatigue is a prominent symptom in chronic liver disease and occurs in a variety of aetiologies including viral hepatitis, cholestatic liver disease or non-alcoholic fatty liver disease [20, 21]. In the current study, the employed questionnaires captured fatigue as the most relevant and burdening symptom in AIH and the CLDQ subdomain fatigue exhibited the lowest score. Similar findings were replicated in a study that employed the modified fatigue impact score (MFIS) and observed a strong impact of AIH on fatigue [14]. A recent study highlighted that fatigue in patients with decompensated liver cirrhosis was associated with anaemia [19]. In the present study, we did not enrol patients with decompensated cirrhosis and no correlation between haemoglobin, haematocrit or erythrocytes and HRQL was detectable (data not shown). More intriguingly, the subscale fatigue was independently affected by the status of biochemical remission. Yet, the mechanisms underlying fatigue in AIH remain poorly understood [22], and further investigations are needed to unveil the underlying pathophysiology. Another aspect is the comparison of mental vs. physical health and a recent study in AIH reported that mental well-being is more severely impaired compared to physical well-being [10].

The EQ-5D-5L was employed to assess general/generic HRQL and the corresponding UI-value can be used to compare it to other disease states. In the EQ-5D-5L, females reported a significantly lower UI-value, as well as a lower score in the subdomain pain/discomfort compared to males indicating that women perceive and experience HRQL impairment stronger than men. Similar findings have been reported previously in other chronic liver diseases [21] and cirrhosis [19] and this is likely related to a different self-perception of the body and general health [23].

Next, we explored surrogate markers of disease activity, as well as clinical parameters and their impact on CLDQ total value and EQ-5D-5L UI-value. On univariable analyses, AST was inversely correlated with CLDQ total value. Elevated AST blood levels have been linked to a progression of disease and treatment failure [24, 25]. Furthermore, patients exhibiting an incomplete biochemical remission had an overall worse CLDQ total value. More notably, using a multivariable approach, AST and incomplete remission were the only independent predictors of an impaired HRQL in this cohort as captured by CLDQ. This could be in parts related to disease progression and its liver-related complications. In fact, a German study previously showed that the severity of depression and anxiety are associated with concerns about disease progression in AIH, consequently leading to a lower HRQL [10]. Furthermore, age did not significantly correlated with CLDQ total value which is in line with findings of a Polish study that did not describe an impact of age using generic HRQL tools [14]. Interestingly, the EQ-5D-5L was more likely to detect non liver related aspects to be associated with a lower HRQL.

Although previous studies have observed a negative impact of corticosteroids on HRQL [12], we did not observe a significant difference between patients receiving corticosteroids vs. no corticosteroids in the current analysis. This is interesting and is most likely related to differences between baseline characteristics and treatment intensity of the two cohorts. Importantly, most of the patients in the current analysis were treated with azathioprine and low dose of corticosteroids or even corticosteroid free, thus providing potential insight on these different findings. However, patients with a complete biochemical remission were lower on steroids, and mostly received an azathioprine monotherapy in comparison to patients with an incomplete remission. Therefore, a lower use of steroids in the complete remission group could likely contribute to an overall lower disease burden due to lower drug-related side effects.

We included a small cohort of 19 patients that were diagnosed with an AIH-overlap syndrome. In this subgroup, HRQL was not significantly different compared to non-overlap patients. These findings differed from observations in the larger analysis in the UK-AIH cohort, when patients with AIH and PBC or PSC overlap exhibited a lower EQ-5D-5L UI-value [12]. These differences can be multifactorial and we have previously observed a country-specific impact between the UK and Germany when exploring HRQL in NAFLD [23].

The current study has several limitations. Most importantly, the two HRQL tools were not specifically validated for AIH. Therefore, the cause for the differences that are captured between the liver-specific tool (CLDQ) and the generic tool (EQ-5D-5L) can only be speculated on. Nevertheless, CLDQ has been widely adopted to study HRQL in different aetiologies of chronic liver disease [26]. In a Japanese study, EQ-5D-5L scores were significantly impaired in AIH patients and were highly associated with corticosteroid use [12]. Importantly and as detailed above, the current analysis was performed in patients that received combined therapy with low-dose steroids. The generalizability of the current study is limited as it constitutes a single-centre analysis and thus the results have to be interpreted with caution. The findings are specific for the outpatient setting at the study centre. Also, HRQL can be impacted by country and culture-specific differences [21] and thus it is difficult to merge data from independent study centres. Yet, one strength of the analysis is the large cohort of 116 AIH patients and the standardized use of two different HRQL questionnaires with one focussing on liver disease and one on general HRQL.

In conclusion, the current study provides evidence that a complete biochemical remission is corresponding to a higher HRQL. The current study identified surrogate markers that correlated with HRQL and can therefore help physicians identify potential HRQL aspects in their patients. However, future studies are warranted to verify these results.

## Supplementary Information

Below is the link to the electronic supplementary material.

Supplementary file1 (DOCX 20 kb)

## Abbreviations

AIH: Autoimmune hepatitis
ALT: Alanine transaminase
ALP: Alkaline phosphatase
AST: Aspartate transaminase
gGT: Gamma-glutamyltransferase
CLDQ: Chronic Liver Disease Questionnaire
EQ-5D-5L: European Quality-of-Life 5-Dimension 5-Level
HRQL: Health-related quality of life
IgG: Immunoglobulin G
IQR: Interquartile range
PBC: Primary biliary cholangitis
PSC: Primary sclerosing cholangitis
