# Supplementary material for: Health-related quality of life in patients with autoimmune hepatitis
Source: Qual Life Res. 2021 May 12;30(10):2853–61. doi: 10.1007/s11136-021-02850-0 (PMC8481193; doi:10.1007/s11136-021-02850-0)
Supplement: Supplementary file 1 — Supplementary file1 (DOCX 20 kb) [file 11136_2021_2850_MOESM1_ESM.docx]

**Supplemental**

**Supplementary Table 1. Differences in Health-Related Quality of Life between male and female**

|  | **Male** | **Female** |  |
| --- | --- | --- | --- |
| **Variable** | (n = 26) | (n = 90) | **p-value** |
| **CLDQ** |  |  |  |
| CLDQ total value | 5.60 (± 0.96) | 5.23 (± 1.31) | 0.28 |
| CLDQ abdominal symptoms | 5.95 (± 1.08) | 5.47 (± 1.60) | 0.33 |
| CLDQ fatigue | 4.50 (± 1.52) | 4.29 (± 1.70) | 0.62 |
| CLDQ systemic symptoms | 5.86 (± 0.96) | 5.34 (± 1.44) | 0.18 |
| CLDQ activity | 6.37 (± 0.89) | 5.69 (± 1.37) | **0.01** |
| CLDQ emotional function | 5.31 (± 1.29) | 5.04 (± 1.41) | 0.38 |
| CLDQ worry | 5.60 (± 1.38) | 5.54 (± 1.43) | 0.95 |
|  |  |  |  |
| **EQ-5D-5L** |  |  |  |
| EQ-5D-5L mobility | 1.27 (± 0.53) | 1.55 (± 0.95) | 0.26 |
| EQ-5D-5L selfcare | 1.07 (± 0.39) | 1.28 (± 0.72) | 0.10 |
| EQ-5D-5L usual activities | 1.46 (± 0.81) | 1.64 (± 0.98) | 0.35 |
| EQ-5D-5L pain/discomfort | 1.58 (± 0.95) | 2.1 (± 0.97) | **0.007** |
| EQ-5D-5L anxiety/depression | 1.57 (± 0.95) | 1.65 (± 0.95) | 0.57 |
| EQ-5D-5L VAS | 71.4 (± 23.1) | 71.2 (± 19.8) | 0.61 |
| EQ-5D-5L UI-Value | 0.90 (± 0.19) | 0.85 (± 0.19) | **0.02** |

Data are expressed as means and standard deviations. Mann-Whitney U test was used to compare CLDQ subscales and total value, as well as EQ-5D-5L VAS, UI-value and its subscales in between groups. Statistical significance is indicated in bold. A p-value < 0.05 was considered statistically significant.

**Supplementary Table 2. Differences in Health-Related Quality of Life between AIH and AIH-Overlap Syndrome**

|  | **AIH** | **AIH-Overlap** |  |
| --- | --- | --- | --- |
| **Variable** | (n = 97) | (n = 19) | **P value** |
| **CLDQ** |  |  |  |
| CLDQ total value | 5.32 (± 1.29) | 5.27 (± 1.03) | 0.56 |
| CLDQ abdominal symptoms | 5.56 (± 1.50) | 5.64 (± 1.53) | 0.86 |
| CLDQ fatigue | 4.42 (± 1.66) | 3.93 (± 1.60) | 0.25 |
| CLDQ systemic symptoms | 5.47 (± 1.39) | 5.36 (± 1.25) | 0.55 |
| CLDQ activity | 5.79 (± 1.25) | 6.10 (± 0.88) | 0.72 |
| CLDQ emotional function | 5.11 (± 1.42) | 5.04 (± 1.19) | 0.65 |
| CLDQ worry | 5.55 (± 1.43) | 5.55 (± 1.38) | 0.95 |
|  |  |  |  |
| **EQ-5D-5L** |  |  |  |
| EQ-5D-5L mobility | 1.52 (± 0.92) | 1.31 (± 0.58) | 0.54 |
| EQ-5D-5L selfcare | 1.28 (± 0.72) | 1.00 (± 0.0) | 0.06 |
| EQ-5D-5L usual activities | 1.62 (± 0.98) | 1.47 (± 0.77) | 0.61 |
| EQ-5D-5L pain/discomfort | 1.98 (± 1.02) | 1.89 (± 0.73) | 0.96 |
| EQ-5D-5L anxiety/depression | 1.68 (± 0.96) | 1.42 (± 0.84) | 0.19 |
| EQ-5D-5L VAS | 71.5 (± 20.1) | 69.7 (± 22.8) | 0.77 |
| EQ-5D-5L UI-Value | 0.85 (± 0.19) | 0.90 (± 0.08) | 0.70 |

Data are expressed as means and standard deviations. Mann-Whitney U test was used to compare CLDQ subscales and total value, as well as EQ-5D-5L VAS, UI-value and its subscales in between groups. Statistical significance is indicated in bold. A P-value < 0.05 was considered statistically significant.

**Supplementary Table 3.** **Comparison of Health-Related Quality of Life between complete and incomplete biochemical remission**

|  | **Complete biochemical remission** | **Incomplete biochemical remission** |  |
| --- | --- | --- | --- |
| **Variable** | **(n = 38)** | **(n = 61)** | **p-value** |
| **CLDQ** |  |  |  |
| CLDQ total value | 5.66 (± 1.15) | 5.10 (± 1.35) | **0.03** |
| CLDQ abdominal symptoms | 6.03 (± 1.38) | 5.36 (± 1.53) | **0.01** |
| CLDQ fatigue | 4.71 (± 1.59) | 4.20 (± 1.72) | 0.15 |
| CLDQ systemic symptoms | 5.84 (± 1.12) | 5.17 (± 1.55) | 0.06 |
| CLDQ activity | 6.15 (± 1.23) | 5.61 (± 1.44) | **0.03** |
| CLDQ emotional function | 5.44 (± 1.40) | 4.88 (± 1.41) | **0.04** |
| CLDQ worry | 5.83 (± 1.47) | 5.34 (± 1.43) | **0.03** |
|  |  |  |  |
| **EQ-5D-5L** |  |  |  |
| EQ-5D-5L mobility | 1.34 (± 0.71) | 1.65 (± 1.10) | 0.18 |
| EQ-5D-5L selfcare | 1.21 (± 0.75) | 1.29 (± 0.79) | 0.66 |
| EQ-5D-5L usual activities | 1.34 (± 0.71) | 1.80 (± 1.20) | **0.03** |
| EQ-5D-5L pain/discomfort | 1.76 (± 0.82) | 2.1 (± 1.10) | 0.26 |
| EQ-5D-5L anxiety/depression | 1.61 (± 0.92) | 1.74 (± 1.10) | 0.68 |
| EQ-5D-5L VAS | 74.9 (± 20.3) | 70 (± 21.3) | 0.21 |
| EQ-5D-5L UI-Value | 0.90 (± 0.13) | 0.83 (± 0.22) | 0.17 |

Patients with complete and incomplete biochemical response and HRQL data are included (exclusion of n=17 due to incomplete labs). Data are expressed as means and standard deviations. Mann-Whitney U test was used to compare CLDQ subscales and total value, as well as EQ-5D-5L VAS, UI-value and its subscales in between groups. Statistical significance is indicated in bold. A p-value of < 0.05 was considered statistically significant.
